# Cell penetrating peptides: a comparative transport analysis for 474 sequence motifs

**DOI:** 10.1080/10717544.2018.1458921

**Published:** 2018-04-14

**Authors:** Katrin Ramaker, Maik Henkel, Thorsten Krause, Niels Röckendorf, Andreas Frey

**Affiliations:** Division of Mucosal Immunology and Diagnostics, Priority Area Asthma & Allergy, Research Center Borstel, Borstel, Germany

**Keywords:** Cell penetrating peptides, combinatorial peptide synthesis, high content screening, cellular uptake, CPP conjugate

## Abstract

Delivering reagents into cells is a key demand in molecular medicine. The vehicle of choice is often cell penetrating peptides (CPPs), which can ferry conjugated cargo across membranes. Although numerous peptides have been shown to promote such uptake events, there has been no comprehensive comparison of individual performance under standardized conditions. We have devised a method to rapidly analyze the ability of a multitude of CPP conjugates to carry a model cargo into HeLa cells. Sequence information for 474 CPPs was collected from literature sources, and the respective peptides were synthesized and modified with carboxyfluorescein (FAM) as model cargo. All candidates were evaluated in an identical uptake test, and transport was quantified using cellular fluorescence intensities. Substantial differences in the ability to carry the fluorophore into the cells were observed, with transport performance differing by a factor of 70 between the best CPP investigated and cargo alone. Strong correlations were observed between uptake efficiency and both sequence length and the presence of positive net charge. A compilation of the 20 top performers with regard to cargo delivery performance and cell compatibility is provided.

## Introduction

Since the discovery that some small cationic peptides are able to transport cargo into living cells (Green & Loewenstein, 1988), a variety of peptides with cell penetrating and cargo transporting abilities has been described (Gautam et al., 2012). Among these peptides are protein derived sequences, e.g. from viral membrane transduction domains (Elliott & O’Hare, 1997), chimeric fusion peptide constructs, for instance transportan sequences (Myrberg et al., 2008), and designed model peptides such as KLA peptides (Scheller et al., 1999). Most of these cell penetrating peptides (CPPs) are cationic (Futaki et al., 2001) or amphiphatic (Deshayes et al., 2011) l-peptides, but rationally designed all-d or mixed l-/d-peptides (Wender et al., 2000) also exist. Not surprisingly for such a variety of different CPP classes, there appears to be no general mode of cellular entry (Madani et al., 2011). Numerous peptides are endocytosed, but others seem to be taken up by living cells in a non-endocytotic manner (Ter-Avetisyan et al., 2009; Bechara & Sagan, 2013). Furthermore, different peptides accumulate in different subcellular compartments including the nucleus (Hariton-Gazal et al., 2002) or cytosol (Delehanty et al., 2010), or remain trapped in intracellular vesicles (El-Sayed et al., 2009). Unfortunately, investigation of the uptake efficiency of CPPs has not been systematic. Tests have been performed using a wide variety of different cell lines, most prominently HeLa cells, Chinese Hamster Ovary (CHO) cells and 3T3 fibroblasts (Gautam et al., 2012). In these tests, the delivery of variant molecular cargoes, such as small molecules, proteins, nucleic acids, or nanoparticles further complicates the situation, making a literature-based comparison of different CPPs extremely difficult (Stalmans et al., 2013).

Since in most experimental studies, the peptides investigated are not compared to a wide variety of other CPPs under identical conditions (e.g. same cell line, cargo, culture and incubation conditions, or detection method), we wanted to devise a method using a defined, reproducible system that would permit the comparison and ranking of a range of CPPs. For this purpose, we selected 474 peptides described as CPPs from the database CPPsite (Gautam et al., 2012), which compiles sequence and structural information of experimentally validated CPPs. The peptides were synthesized and were then equipped with a fluorophore model cargo, before being tested under identical conditions in a cell-based assay setup. In contrast to another method for the comparison of CPPs described by Stalmans et al. (2013), which was based on multivalent data-analysis of 186 known CPPs, our approach provides the opportunity to directly evaluate and compare cell penetrating properties of peptides in a defined yet modifiable setup.

## Materials and methods

### Peptide synthesis

Peptides were synthesized by Fmoc solid phase synthesis techniques using standard amino acid building blocks on an automated multiple peptide synthesizer (MultiPep RS, Intavis Bioanalytical Instruments AG, Cologne, Germany). Peptides were assembled on an amide resin (TentaGel S RAM 0.24 mmol/g, Rapp Polymers, Tübingen, Germany), 1.3 g of which was swollen in 50 ml of a 7:3 mixture of dichloromethane (DCM, Roth, Karlsruhe, Germany) and dimethylformamide (DMF, Biosolve Chemicals, Valkensvaard, Netherlands) and transferred to a 96-well filter bottom microplate (96-well reaction plate 2–5 µmol, Intavis Bioanalytical Instruments AG) in portions of about 3 µmol per well. The resin was primed for synthesis by washing three times with 300 µl of DMF per well. Fmoc deprotection was accomplished by treating the resin twice for 5 min with 170 µl of a mixture of 20% (v/v) piperidine (Sigma-Aldrich, Steinheim, Germany) in DMF and subsequent washing seven times with 250 µl of DMF per well. Coupling was achieved by reacting a 10-fold molar excess of Fmoc protected amino acid building blocks (Merck, Darmstadt, Germany; IRIS Biotech, Markdrewitz, Germany) with the resin twice for 30 min each at room temperature (RT). For this, each time a mixture of 24 µl of a 0.6 M solution of Fmoc amino acid building block in DMF, 8 µl of a 4 M solution of 4-methylmorpholine (NMM, Sigma-Aldrich, Steinheim, Germany) in DMF, and 23 µl of a 0.6 M solution of 2-(1H-benzotriazol-1-yl)-1,1,3,3-tetramethyluronium hexafluorophosphate (HBTU, IRIS Biotech, Marktredwitz, Germany) in DMF was added per well. After washing three times with 300 µl of DMF per well, unreacted amino termini were capped for 5 min with 120 µl of a 5% (v/v) mixture of acetic anhydride (Merck, Darmstadt, Germany) in DMF per well at RT. Subsequently, the resin was washed six times with 250 µl of DMF per well. After coupling of the last amino acid, an additional coupling round under identical conditions was performed using 5(6)-carboxyfluorescein (FAM, Merck, Darmstadt, Germany) instead of an amino acid building block to attach the fluorophore cargo to the peptides at the N-terminus.

After synthesis, the resin was treated three times for 5 min with 170 µl of a solution of 20% (v/v) piperidine in DMF and then was washed 10 times with 250 µl of DMF per well. Amino termini were subsequently capped twice for 5 min with 120 µl of a 5% (v/v) mixture of acetic anhydride in DMF per well at RT, then the resin was washed eight times with 250 µl of DMF and additional six times with 150 µl of DCM per well. DCM was removed and the resin was dried *in vacuo* for 12 h at RT. Peptides were cleaved from the resin by incubating it three times with 100 µl of cleavage cocktail (92.5% of trifluoroacetic acid (TFA, Roth, Karlsruhe, Germany), 5% of triisobutylsilane (TIBS, Sigma-Aldrich, Steinheim, Germany) and 2.5% of water (v/v)) per well for 10 min, 30 min, and 90 min at RT. After each incubation, the cleavage cocktail was collected well-wise in a 96-well plate (MegaBlock 96 well 2.2 ml, Sarstedt, Nümbrecht, Germany). The peptide containing solutions were treated with 1500 µl of a 1:1 mixture of cold *tert*-butyl methyl ether (TBME, Sigma-Aldrich, Steinheim, Germany) and cyclohexane (Sigma-Aldrich, Steinheim, Germany) per well. The precipitated peptides were separated by centrifugation (2800×*g*, 5 min, Heraeus Megafuge 1.0R, Hanau, Germany) and the supernatants were removed; this step was repeated three times with 1500 µl of cold TBME. The residues were dissolved in 500 µl of a mixture of acetic acid, acetonitrile and water (1:2:2) and dried *in vacuo*.

### Adjustment of peptide concentrations

A total of 1 ml of a 1:1 mixture of acetonitrile (VWR Chemicals, Radnor, PA) and water was added to each well of the 96-well plates containing the dried peptide conjugates. The plates were treated in an ultrasound bath for 20 min at 40 °C; insoluble matter was removed by centrifugation (2800×*g*, 5 min) and 2 × 180 µl from each well were transferred to two 96-well polystyrene plates (Corning, Amsterdam, Netherlands). In one plate, 20 µl of acetic acid (Roth, Karlsruhe, Germany) were added to each well, the OD_490_ was measured in a plate reader (SpectraMax M5, Molecular Devices, Sunnyvale, CA), and the concentrations of the peptide solutions were determined according to a calibration curve using FAM in 45% acetonitrile/45% water/10% acetic acid as standard. Then, 20 µl from each well of the second plate were transferred to micro reaction vessels and the peptide solutions were diluted with the calculated amounts of 20% ethanol (v/v) in water in order to obtain 50 µM stock solutions of peptide/FAM conjugates.

### Cell culture

The human cervix adenocarcinoma cell line HeLa was obtained from I.A.Z. (Munich, Germany). As cell growth medium DMEM/Ham’s F-12 (1:1) supplemented with 2 mM l-glutamine, 10% fetal bovine serum, and 1% penicillin/streptomycin (all Capricorn Scientific, Ebsdorfergrund, Germany, formerly PAA Laboratories) was used, and cells were cultured at 37 °C and 5% CO_2_. They were passaged before reaching confluence; for the experiments, cells at passages p8–p20 were used. For testing uptake efficiency and cytotoxicity of CPPs, cells were seeded in 96-well µ-plates (ibidi, Munich, Germany) at a concentration of 6 × 10^4^ cells per well and incubated for 48 h to reach confluence before performing the experiments.

### CPP uptake experiments

All solutions used were warmed to 37 °C, with CPP incubation performed at 37 °C and 5% CO_2_. CPP working solutions were prepared in 96-well polypropylene plates (Corning, Amsterdam, Netherlands) directly before incubation by diluting the 50 µM FAM-labeled CPP stock solutions to 10 µM with cell growth medium. The confluent HeLa cells in the 96-well µ-plates were exposed to 100 µl of a 10 µM CPP working solution per well for 90 min. Cell growth medium served as negative control. After incubation, the cells were washed twice with 300 µl of CM-PBS (PAA Laboratories), then 100 µl CM-PBS was added before determining fluorescence intensities in a plate reader (SpectraMax M5, Molecular Devices, Sunnyvale, CA) at an excitation wavelength of 495 nm and an emission wavelength of 530 nm. Transmission images of the treated cell layers were taken using a MORE microscope (TILL Photonics, Munich, Germany) equipped with a 10×/0.45 objective (Zeiss, Jena, Germany), an FITC filterset (ET480_40x/ET535_50 m/T510LPXRXT, Chroma, Rockingham, NC) and LED and oligochrome light source (FEI). Images were acquired using a Stingray F-145B CCD camera (Allied Vision Tech., Stadtroda, Germany) via live acquisition software (v2.2.2, FEI).

### Cytotoxicity MTT test

HeLa cells were treated with 100 µl of 10 µM FAM-labeled CPPs in cell growth medium as in the CPP uptake experiments. After CPP incubation, 10 µl of MTT (thiazolyl blue tetrazolium bromide, Sigma-Aldrich, Steinheim, Germany) solution (2.5 mg/ml in cell growth medium) were added, and the cells were incubated for another 2 h at 37 °C and 5% CO_2_. Thereafter, 290 µl of extraction solution (93.1% isopropanol (Sigma-Aldrich, Steinheim, Germany) and 6.9% formic acid (Sigma-Aldrich)) were added to reach a final volume of 400 µl fluid containing 5% formic acid. Subsequently, the cells were treated for 5 min at RT in an ultrasound bath. After cell lysis and centrifugation (3350×*g*, 5 min, Heraeus Megafuge 1.0R), 200 µl of the supernatant were transferred to a 96-well polystyrene plate (Corning) and the OD_570_ was determined using a plate reader (SpectraMax M5). HeLa cells incubated in cell growth medium without CPP were defined as 100% viable.

### Statistics

Between group comparisons were performed using GraphPad Prism v5 (GraphPad Software, La Jolla, CA). Differences were assessed by the Kruskal–Wallis test, with Dunn's Multiple Comparison as post hoc test. Results with *p* < .05 were considered significant.

## Results

### Selection and synthesis of CPPs

To select CPPs for our experiments, we used the database CPPsite (Gautam et al., 2012), which at the time of our query listed 741 unique peptide sequences with described cell penetration capability. We excluded peptides longer than 24 amino acids or bearing non-proteinogenic amino acid residues, cyclic peptides, and CPPs with receptor-mediated or pore-forming uptake mechanisms. Based on these criteria, a final selection of 474 peptides between 4 and 24 amino acids in length remained as our working pool of CPPs (Supplementary Table TS1).

All peptides were synthesized by solid phase peptide synthesis under rigorous coupling conditions to minimize truncation of peptides and to ensure that only correct full-length peptides were equipped with the cargo of interest. This included a double coupling procedure with 10-fold excess of fluorenylmethyloxycarbonyl (Fmoc) protected amino acid monomers in each synthesis cycle, with strict capping steps to block any remaining free amino functions after each coupling cycle. In the last step of the synthesis, all 474 peptides were equipped N-terminally with a carboxyfluorescein (FAM) moiety as model cargo. This FAM residue not only represents the cargo but also acts as a reporter group (fluorophore) in the biological assay system. Our synthesis procedure guarantees that only completely synthesized peptides can carry the fluorophore and hence give a fluorescence signal in biological assay systems; truncated sequences are capped during synthesis and therefore lack the carboxyfluorescein moiety. To verify the identity of the synthesized CPPs, 10% of all peptides were randomly selected and checked by mass spectrometry; the expected products were confirmed in all preparations analyzed (Supplementary Table TS2).

### Concentration adjustment of CPPs

For the parallel investigation of cell-peptide interactions in the 96-well plate-based assay, a convenient method for standardization of CPP concentrations was developed. This method had to allow reliable concentration adjustment without stressing the cells with a high content of organic solvent in the CPP working solutions. As the solubility of peptides in aqueous buffer is strongly related to their amino acid sequence, it was necessary to use organic solvent to enable complete dissolution of all peptides of interest. Therefore, all FAM-labeled peptide derivatives were dissolved in 50% acetonitrile/water (v/v). For standardization, one aliquot of each peptide solution was supplemented with acetic acid to a final concentration of 10% (v/v) which lowered the pH to 2.4 and peptide concentrations were quantified by determining the optical density (OD) at 490 nm. A pH of 2.4 was selected because the extinction coefficient of FAM is drastically reduced at low pH, and therefore high CPP concentrations could only be measured photometrically with appropriate dynamic signal range under these conditions. FAM dissolved in the same solvent as the peptides was used as the standard for the calibration curve (Supplementary Figure S1). The peptide solutions in acetonitrile/water were then diluted with 20% of ethanol/water (v/v) according to their OD in order to obtain 50 µM stock solutions. Ethanol was chosen in this step as solubility enhancer since it is far less toxic in subsequent cell assays than acetonitrile.

To verify uniformity of the concentration-normalization of the peptide stock solutions, the 50 µM stocks were further diluted to 20 µM with PBS and the OD was measured at the isosbestic point of fluorescein (460 nm), a wavelength where the pH dependence of the molar extinction coefficient is lowest (Thomas et al., 1979) (Supplementary Figure S2). Here, the mean ± standard deviation OD of all stock solutions was 0.157 OD_460_ ± 0.036 OD, which translates into a coefficient of variation (CV) of approximately 23%.

### Uptake of CPPs into HeLa cells

HeLa cells are most frequently used as a model system for testing the uptake efficiency of CPPs for human cells. Indeed, more than one-third of the CPPs described in the literature have been validated for their cell penetrating capability using HeLa cells (Gautam et al., 2012), making these cells a favorable model cell line for our study. As the standard concentration for all CPPs in our comparative cell uptake assays, we chose 10 µM, which had also been widely used in other studies (Mueller et al., 2008). At this concentration, the cells are unlikely to be substantially damaged by the CPPs as indicated by MTT assays (Mosmann, 1983; Mueller et al., 2008), and consequently the uptake efficiency of the CPPs can be judged by quantifying the intracellular fluorescence of the FAM cargo.

Tests for comparative analysis of a multitude of different peptides from a library are usually carried out in an array-based solid phase format (Röckendorf et al., 2007), but for the investigation of the interactions of different peptides with living cells, plate-based assay formats are more suitable. To set up a cell-based assay system capable of comparing a large number of different CPPs, the cells were grown in 96-well µ-plates, which provide a surface for proper cell growth while also allowing visualization of the cells in a microscope to check their integrity after exposure to CPPs. Culturing conditions of the HeLa cells were optimized to reproducibly obtain a confluent cell layer 48 h after seeding. Confluent cell layers should reflect the *in vivo* situation at epithelial surfaces in the best possible way.

**Figure 1.**
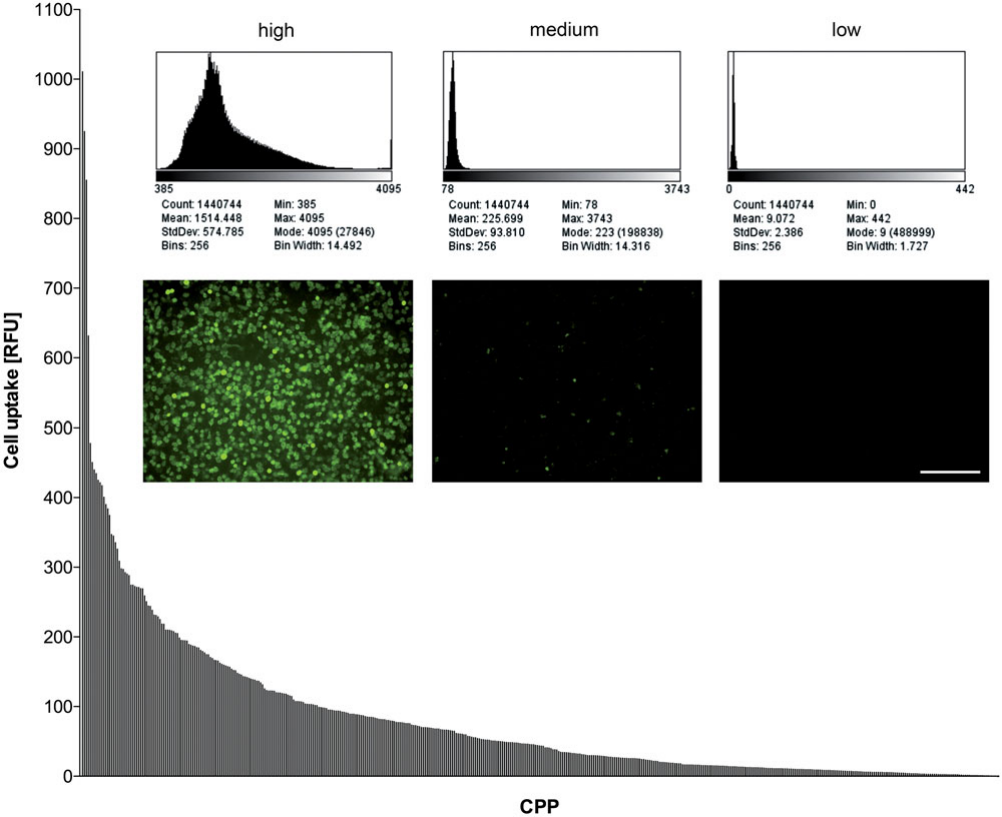
Fluorescence intensities (RFU) of HeLa cells after incubation with 10 µM FAM-labeled CPPs. The peptides are sorted by signal intensities, with bars indicating the mean of two measurements. The inset shows examples of the performance of one CPP candidate each of high, medium and low uptake efficiency (fluorescence images of the cell layers with histograms detailing the respective brightness of untreated 12-bit images). Bar: 200 µm.

Exposing those confluent HeLa cell monolayers to the 474 different FAM-conjugated peptides revealed dramatic differences in transport efficiency (Figure 1). While some CPP motifs did not aid FAM uptake at all, the best performers facilitated FAM entry into HeLa cells by a factor of 70 compared to FAM alone. A summary of the performance of the top 20 CPPs is given in Table 1. Without being coupled to a CPP ferry, FAM was taken up to 14.3 ± 8.1 relative fluorescence units (RFU; mean value and standard error of two measurements) into the HeLa layer.

**Table 1.** Characteristics and uptake performance of the top 20 CPPs in our setup.

	Sequence	Fluorescence (RFU)	Sequence length	Net charge at pH 7.4	Hydropathy score	Uptake mechanism and localization*^,^#	Category
1	WLRRIKAWLRRIKALNRQLGVAA	1011.42	23	+7	0.3	Unknown	Amphipathic†
2	VKRKKKPALWKTLLKKVLKA	925.42	20	+9	12.0	a/c	Amphipathic†
3	KTVLLRKLLKLLVRKI	855.56	16	+6	2.0	Unknown	Amphipathic†
4	KKKKKKKKKKKKKKKKKKK	632.01	19	+19	57.0	Unknown	Amphipathic†
5	KLALKLALKALKAALK	478.40	16	+5	1.7	a/d	Amphipathic#
6	RQARRNRRRALWKTLLKKVLKA	450.97	22	+10	16.4	a/d	Amphipathic†
7	KLALKLALKALKAALKLA	440.41	18	+5	–0.6	a/d	Amphipathic#
8	LLKKRKVVRLIKFLLK	435.30	16	+7	4.7	Unknown	Amphipathic†
9	LIRLWSHLIHIWFQNRRLKWKKK	425.60	23	+9	–4.6	b/c	Amphipathic#
10	LNSAGYLLGKINLKALAALAKKIL	422.13	24	+4	–8.3	b/e	Amphipathic#
11	CWKKKKKKKKKKKKKKKKKK	417.94	20	+18	49.6	Unknown	Cationic#
12	YTAIAWVKAFIRKLRK	401.31	16	+5	–2.0	Unknown	Amphipathic†
13	PKKKRKVALWKTLLKKVLKA	390.81	20	+9	12.0	a/d	Amphipathic†
14	GLWRALWRALRSLWKLKRKV	384.64	20	+7	–0.4	Unknown	Amphipathic†
15	GLWRALWRGLRSLWKKKRKV	375.17	20	+8	4.9	Unknown	Amphipathic†
16	GLWRALWRGLRSLWKLKRKV	347.66	20	+7	0.1	Unknown	Amphipathic†
17	KALAKALAKLWKALAKAA	345.58	18	+5	0.4	a/d	Amphipathic#
18	KLAAALLKKWKKLAAALL	335.89	18	+5	–2.2	a/d	Amphipathic#
19	GLFKALLKLLKSLWKLLLKA	326.66	20	+5	–7.8	Unknown	Amphipathic#
20	KLALKLALKAWKAALKLA	309.34	18	+5	–2.2	a/d	Amphipathic#

Fluorescence values are given for HeLa cells after incubation with 10 µM FAM-labeled CPP (mean of two experiments).

* a: non-endocytic pathway; b: endocytic pathway; c: cytoplasm; d: cytoplasm and nucleus; e: cytoplasm and nucleus and intracellular membranous structures.

# According to the database CPPsite.

† According to the predicted secondary structure at database CPPsite.

**Figure 2.**
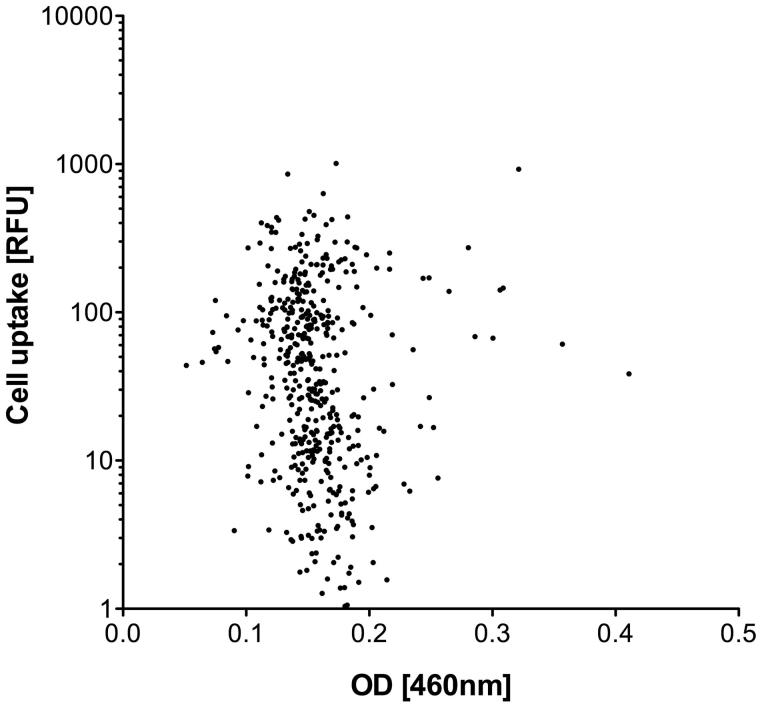
Uptake efficiency (RFU) of FAM-CPPs into HeLa cells (see Figure 1) in relation to the final peptide concentration measured at the isosbestic point. The final peptide concentration is represented by the optical density (OD) at a wavelength of 460 nm after dilution of the stock solutions with PBS.

To rule out the possibility that variations in the normalization procedure caused or contributed to these uptake differences, transport efficiency as indicated by the fluorescence intensity (RFU) of the FAM-CPP exposed cell layers was set in relation to the final peptide concentrations measured at the isosbestic point (Figure 2). No correlation was found between these two parameters, demonstrating that the variations that occurred during concentration-normalization did not cause the differences in the uptake rate. Consequently, other reasons endogenous to the CPP sequence motif must account for the differences observed.

### Influence of sequence length, net charge and hydropathy score on CPP uptake efficiency

**Figure 3.**
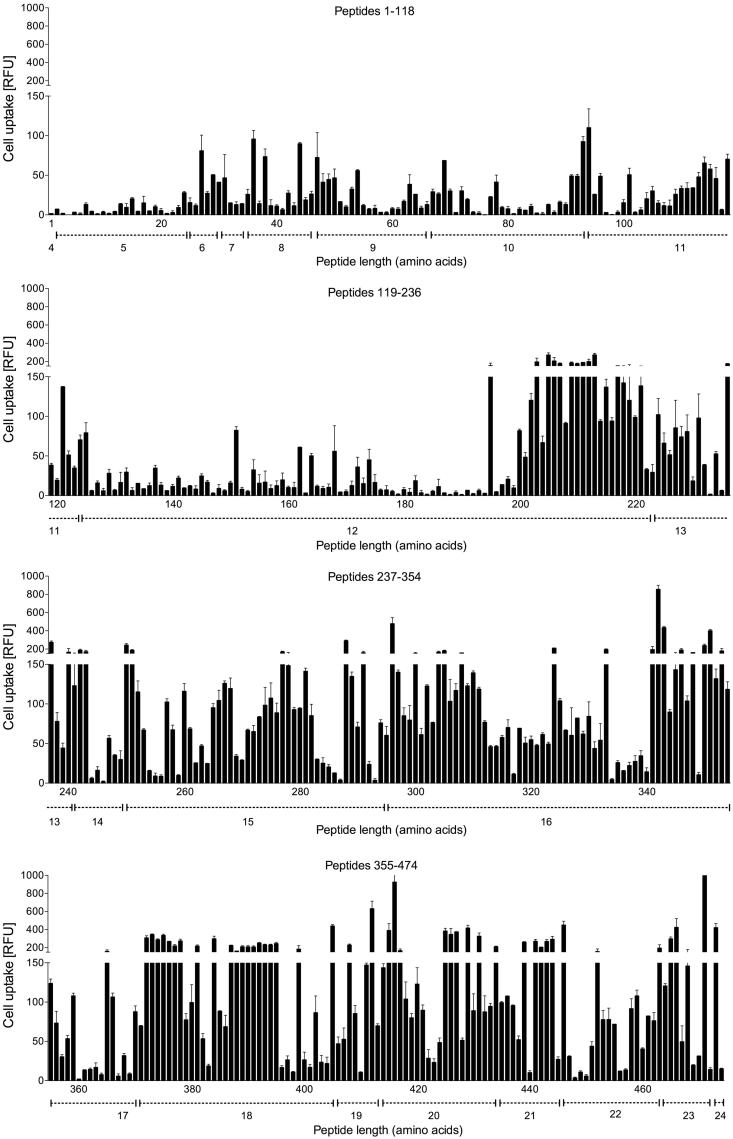
Fluorescence intensities (RFU) of HeLa cells after incubation with 10 µM FAM-labeled CPPs. The peptides are sorted by sequence length, in the same order as listed in Supplementary Table TS1. Bars indicate mean value and standard error of two measurements, dotted lines under the diagrams delineate ranges of the different peptide lengths (4–24 amino acids).

**Figure 4.**
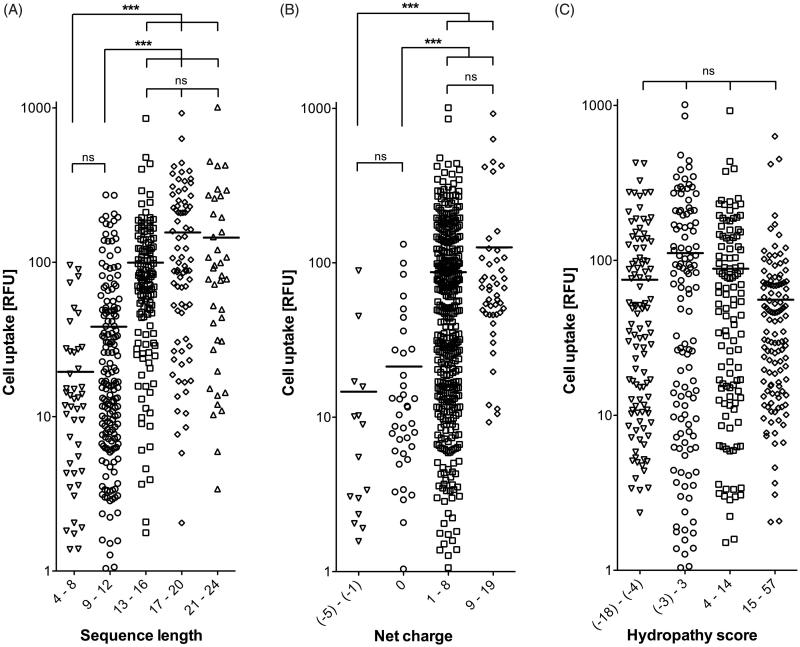
Cell uptake of the CPPs (expressed in fluorescence intensities (RFU)) compared with respect to sequence length (A), net charge (B), and hydropathy score (C). Horizontal lines indicate the mean value of each group. Differences between groups were assessed for statistical significance by the Kruskal–Wallis test: ***p < .001; ns: not significant.

Therefore, we took a closer look at the uptake efficiency of the CPPs in relation to sequence length. To visualize possible effects of sequence length, the fluorescence intensities of HeLa cells after exposure to the 474 FAM-labeled CPPs were sorted by peptide length (Figure 3). This depiction shows that peptide length was an important factor influencing uptake, since peptides which were below 13 amino acid residues in length showed significantly (*p* < .0001) lower uptake levels than those composed of 13 or more amino acids. No significant differences could be observed within the group of shorter (4–8 vs. 9–12 amino acids) or longer peptides (13–16 vs. 17–20 vs. 20–24 amino acids) (Figure 4(A)). Thus, a certain peptide length seems to be a necessity for good performance, although not the sole requirement, since even among the longest motifs tested, poor performers were present (Figure 3).

Another important parameter in the CPPs sequences was found to be the net charge at physiological pH 7.4 (Supplementary Table TS1), as calculated using a generic formula (Supplementary Formula F1). Sequences with a positive net charge were internalized significantly better (*p* < .0001) than those with either negative or no net charge at physiological pH (Figure 4(B)). Since there were no significant differences between slightly and highly positively charged peptides (net charges of 1–8 vs. 9–19), the cellular uptake of CPPs is unlikely to depend solely on the amount of positive charges. A third criterion could be the lipophilicity of a peptide sequence motif (Zhang et al., 2014). To address that question, the hydropathy scores of the peptides (Supplementary Table TS1) were generated according to Hopp & Woods (1981) by assigning each amino acid a hydrophilicity value (Supplementary Table TS3) and then calculating the total hydropathy using Supplementary Formula F2. However, when comparing the peptides according to their hydropathy scores, no significant differences in the uptake performances of the four groups could be found (Figure 4(C)).

### Integrity of cell monolayers and viability of cells after incubation with CPPs

**Figure 5.**
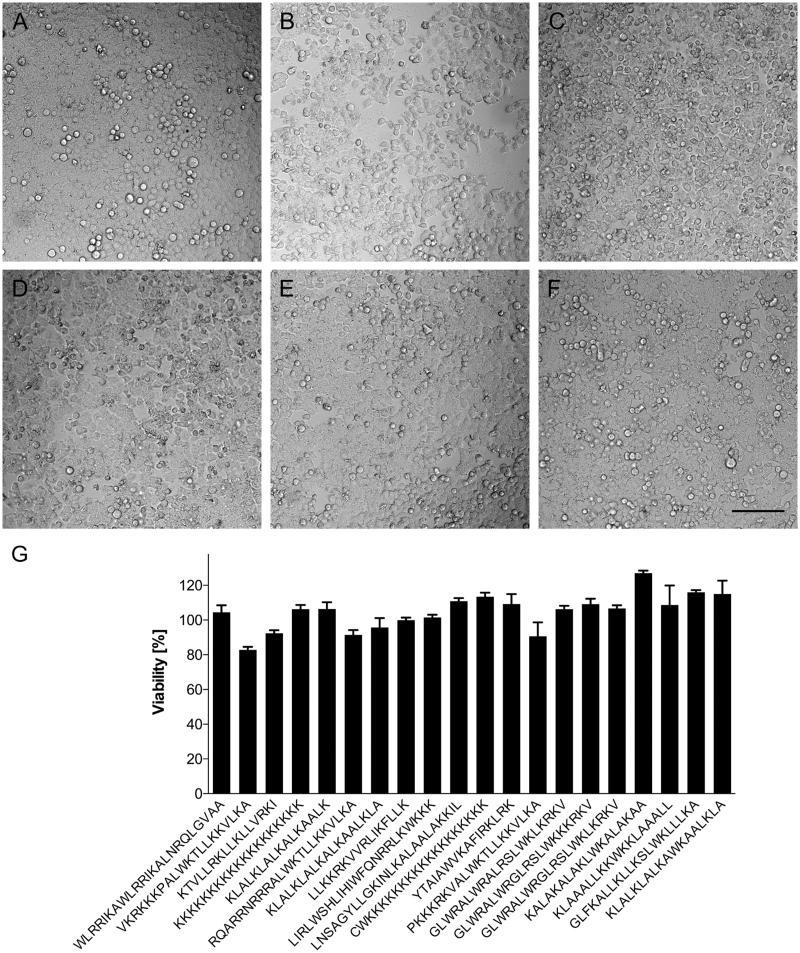
Viability of HeLa cells after incubation with 10 µM of FAM-labeled CPPs for 90 min at 37 °C. (A–F) En face microscopic analysis of cell layers after incubation with: (A) medium only; (B) WLRRIKAWLRRIKALNRQLGVAA; (C) VKRKKKPALWKTLLKKVLKA; (D) KTVLLRKLLKLLVRKI; (E) KKKKKKKKKKKKKKKKKKK; and (F) KLALKLALKALKKAALK. Dark shadows are caused by proximity to well walls. Bar 100 µm. (G) Cell viability, analyzed by MTT-test, after incubation with top 20 CPP in our setup (see Table 1; 1–20 from left to right). Bars indicate mean values and standard deviations of quadruplicate measurements. HeLa cells incubated in cell growth medium without CPP were defined as 100% viable.

Carrying cargo into a cell may cause harm to the membrane crossed. We therefore wanted to know whether effective carriers are able to enter a cell without compromising its viability. For that reason, the integrity of the confluent HeLa cell layers was monitored routinely by phase contrast microscopy prior and postincubation with candidate FAM-CPPs, using culture medium as control. Figure 5 shows images of the top five CPPs in terms of uptake. The confluent cell layer remained intact when performing the experiments with medium (Figure 5(A)); the candidate peptides ranked 4 and 5 (Figure 5(E): KKKKKKKKKKKKKKKKKKK; Figure 5(F): KLALKLALKALKAALK) also barely affected the morphology of the monolayer. However, the CPPs ranked 1–3 (Figure 5(B): WLRRIKAWLRRIKALNRQLGVAA; Figure 5(C): VKRKKKPALWKTLLKKVLKA; Figure 5(D): KTVLLRKLLKLLVRKI) led to detachment of some cells from the plate, indicating a connection between CPP uptake and cell morphology. Nevertheless, the viability of the cells was not compromised by changes in cell morphology, with viability largely unaffected after 90 minutes of incubation with CPPs for the top 20 peptides as verified by MTT assay (Figure 5(G)).

## Discussion

The aim of this study was to identify the most effective CPPs for a given transport task among the CPPs so far reported. We developed a methodology to rapidly compare a range of peptides described as CPPs, using a defined experimental setup. To permit this, we first devised a procedure that allowed the normalization of the concentration of the synthesized peptides. Since the synthesized CPPs were equipped with their FAM cargo at the amino terminus, only complete CPPs were labeled, and we therefore decided to utilize the optical properties of FAM for the adjustment of concentrations via measurement of the peptide solutions’ OD at 490 nm. Here, it is important to keep the pH of the various solutions within a narrow range, to ensure the formation of comparable prototropic forms of FAM, since variant protonations can impact their spectral properties (Klonis & Sawyer, 1996). Using this approach, peptide concentrations could be normalized within a CV of about 23%. As measurements yielding CVs above 15% should be treated with some caution, we further checked for concentration independence of the FAM-CPPs, by relating uptake efficiency to OD. No correlation could be established, indicating that eventual differences in uptake efficiency must be due to physicochemical differences among FAM-CPP conjugates.

Such differences in uptake efficiency were indeed detectable. CPP transport performance ranged from nonexistent, i.e. the uptake rate of FAM-CPP and FAM alone were the same, to improving cargo uptake by a factor of 70. Influence of organelle-specific pH variance on intracellular FAM fluorescence should contribute little to that, if at all. For the Golgi network, early and recycling endosomes, peroxisomes, nucleus, cytosol, endoplasmatic reticulum, and mitochondria, whose pH ranges from 6 to 8 (Casey et al., 2010), FAM fluorescence can vary by a maximum of 30% depending on the pH (see Supplementary Figure S2). Only in the cellular disposers (secretory granules, late endosomes, and lysosomes), where the pH is below 6, FAM fluorescence will be compromised to a larger extent and yield unfavorable readouts for respective CPPs. However, CPPs accumulating at those sites would be of little interest anyway.

A range of 70 in cargo delivering capacity caused by the peptide alone is considerable, yet not implausible if one takes into account that we have compared CPP candidates which had emerged from different studies. As every study is unique with regard to experimental setup, cargo, cell line, culture conditions and peptide quality, a sequence motif presented as top performer in one setup may turn out to be mediocre under different conditions. On the other hand, peptides originating from the same ‘sequence family’, e.g. the pVEC peptides derived from the murine vascular endothelial cadherin protein (Elmquist et al., 2001) or derivatives of the amphiphatic peptide carrier MPG (Crombez et al., 2009), displayed comparable abilities to carry FAM into HeLa cells in our defined setup. This argues in favor of the robustness of our assay system and the validity of our ranking.

Yet, no assay system is able to yield all the information that may be wished for. In our setting, a delicate problem that is difficult to resolve remains the discrimination between adherent and taken up CPPs. As adherence is prerequisite for uptake one captures a snapshot of an ongoing process as long as equilibrium has not been reached. However, even at equilibrium all stages of the uptake process should be visible. In order to get as close as possible to the final CPP distribution, we set the incubation time for the CPP peptides to 90 min which is 1.5-fold longer than the maximum time span reported to reach steady state (Delaroche et al., 2007; Jiao et al., 2009). Thus, it is likely that our data represent the equilibrium stage or a situation very close to that but it may be inevitable to detect some adhesion on top of the CPP uptake.

In our assay, we observed a statistically significant dependence of CPP uptake efficiency with both net charge and peptide length. Peptides with positive net charge penetrated the cell membrane significantly better than ones with negative or no net charge. An explanation for the more effective uptake of positively charged CPPs may lie in the existence of negatively charged proteoglycans and phospholipids on the cell surface that enable an electrostatic interaction between positively charged CPPs and the membrane as first step for internalization, followed by endocytic pathways or direct translocation (Bechara & Sagan, 2013). Various models exist to explain such a direct translocation of a peptide across the cellular lipid bilayer, via inverted micelle formation, adaptive translocation or pore formation (Bechara & Sagan, 2013). Positively charged CPPs can be divided into amphipathic peptides and cationic peptides that do not form an amphipathic α-helix (Milletti, 2012). Most of our top CPPs are amphipathic α-helical peptides (Table 1) according to the predicted secondary structure at the CPP site described by Gautam et al. (2012). Furthermore, CPPs above 12 amino acid residues in sequence length show significantly higher uptake levels in our test system than shorter peptides. A correlation between the sequence length of peptides and their uptake efficiency has already been found for KLA peptides (Scheller et al., 1999). The authors of that study suggested that a peptide length corresponding to four helix turns is essential for efficient cellular uptake. Our top α-helical CPPs consist of 16–23 amino acids and therefore fulfill this requirement. As the helical structure of short peptides in solution is very dynamic, we speculate that peptides below 13 amino acids in sequence length do not exist in α-helical conformation to a sufficient extent to be taken up by the cells. Moreover, 13mer α-helical peptides would be about 1.65 nm in length, sufficient to penetrate the layer of fully hydrated polar head groups of the cellular plasma membrane, thus reaching the dehydrated alkyl core of the lipid bilayer. It is speculated in the literature that amphiphilicity is more important for membrane translocation than the positive charge of the peptide (Milletti, 2012). In the α-helix of such amphipathic CPPs, the hydrophobic and the hydrophilic amino acids are grouped, forming a positively charged and an apolar surface as visualized by helical wheel notation of peptide sequences. These findings should be investigated in more detail, but may potentially facilitate more precise design of CPPs. A mere high lipophilicity of a sequence motif seems not to be sufficient to promote transport.

On top of the inherent properties that enable a CPP to enter mammalian cells *in vitro*, it should be kept in mind that a CPP-based/-enhanced drug delivery system has to cope with more variables than target cell surface properties when applied *in vivo*. Here, factors such as unfavorable distribution in the body, adverse effects beyond that tested in this study and premature excretion or breakdown may interfere with CPP-mediated intracellular drug delivery (Amantana et al., 2007; Sarko et al., 2010).

In light of all of the above, it is certainly not possible to identify one ‘ultimate’ CPP for all purposes. Yet, a general trend for a ‘good performer’ in our set up can be deduced when comparing the characteristics of our top CPPs (Table 1): such a candidate should exhibit a length above 15 amino acids, display a positive net charge, and form an amphipathic α-helix.

Among the peptide candidates with the highest fluorescence signals in our setup, the best performers were: the permeant MAPKAP kinase 2 inhibitory peptide MK2i (WLRRIKAWLRRIKALNRQLGVAA) (Lopes et al., 2010); a CPP derived from the antimicrobial peptide dermaseptin S4 attached to a nuclear localization signal (VKRKKKPALWKTLL-KKVLKA) (Hariton-Gazal et al., 2002); and the designed model peptides E162 (KTVLLRKLLKLLVRKI) (Hällbrink et al., 2005), polylysine 19 (KKKKKKKKKKKKKKKKKKK), and peptide III (KLALKLALKALKAALK) (Scheller et al., 1999).

## Conclusions

Taken together, our study reveals that the most important general properties of CPPs are length and net charge of the peptides. Peptide length is obviously associated with the ability of longer peptides to form more ordered, probably α-helical structures. A positive net charge is definitely beneficial, albeit no significant differences between slightly and highly positively charged peptides were found. FAM transport performance differed significantly among the candidates tested. Importantly, we clearly demonstrated the value of conducting an assay such as the one described here to identify the best performing CPP sequence motifs for a given task.

## Supplementary Material

Ramaker_et_al_Supplementary_Material_revised.pdf
