# Supplementary material for: Cell penetrating peptides: a comparative transport analysis for 474 sequence motifs
Source: Drug Deliv. 2018 Apr 14;25(1):928–37. doi: 10.1080/10717544.2018.1458921 (PMC6058608; doi:10.1080/10717544.2018.1458921)
Supplement: Ramaker_et_al_Supplementary_Material_revised.pdf [file IDRD_A_1458921_SM6422.pdf]

Supplementary Material for

Cell penetrating peptides: a comparative transport analysis for 474 sequence motifs

Katrin Ramaker, Maik Henkel, Thorsten Krause, Niels Röckendorf and Andreas Frey.

Division of Mucosal Immunology and Diagnostics, Priority Area Asthma & Allergy, Research Center Borstel, Airway Research Center North (ARCN), Member of the German Center for Lung Research (DZL), Parkallee 22, 23845 Borstel, Germany.

Supplementary Table TS1

Peptides synthesized and tested for their uptake efficiency in live HeLa cells. Net charge and hydropathy score are calculated according to Supplementary Formulae F1 and F2 (see below).

No.	Sequence	RFU 1	RFU 2	Mean RFU	Net charge pH 7.4	Hydro-pathy score
1	YRFK	1.82	1.82	1.82	2	1.2
2	RRRRR	7.53	7.19	7.36	5	15.0
3	VPMLK	1.96	2.15	2.05	1	-1.6
4	VPTLK	-0.25	-1.17	-0.71	1	-0.7
5	VPALR	3.43	3.52	3.48	1	-0.8
6	VSALK	2.70	0.05	1.38	1	-0.5
7	PMLKE	15.12	11.44	13.28	0	2.9
8	VPALK	4.91	3.92	4.42	1	-0.8
9	VSLKK	1.81	0.98	1.39	2	3.0
10	VSGKK	2.56	4.61	3.59	2	4.8
11	KLPVM	2.32	1.17	1.74	1	-1.6
12	IPMIK	4.80	3.80	4.30	1	-1.9
13	KLGVM	12.94	14.48	13.71	1	-1.6
14	KLPVT	14.21	4.83	9.52	1	-0.7
15	VPMIK	18.74	22.01	20.37	1	-1.6
16	IPALK	4.88	3.68	4.28	1	-1.1
17	IPMLK	23.55	6.85	15.20	1	-1.9
18	VPTLQ	4.68	5.23	4.96	0	-3.5
19	QLPVM	12.28	8.44	10.36	0	-4.4
20	ELPVM	2.84	8.17	5.51	-1	-1.6
21	VPTLE	2.11	1.70	1.91	-1	-0.7
22	EEEAA	0.97	5.16	3.06	-3	8.0
23	IGCRH	11.86	7.14	9.50	2	-0.3
24	CWKKK	26.80	29.48	28.14	3	4.6
25	RRRRRR	21.47	9.55	15.51	6	18.0
26	MIIYRD	9.88	13.71	11.80	0	-1.2
27	FIRIGC	100.65	61.22	80.93	1	-4.1
28	PFVYLI	29.44	25.15	27.29	0	-9.9
29	RIFIGC	51.00	49.94	50.47	1	-4.1
30	RRRRRRR	41.18	41.42	41.30	7	21.0
31	RRMKWKK	76.20	17.32	46.76	5	10.3
32	PKKKRKV	14.66	15.49	15.08	5	13.5
33	FKKFRKF	16.50	9.51	13.00	4	4.5

No.	Sequence	RFU 1	RFU 2	Mean RFU	Net charge pH 7.4	Hydro-pathy score
34	MIIYRDL	14.51	13.46	13.99	0	-3.0
35	RKKRRQRR	32.43	19.51	25.97	7	21.2
36	RRRRRRRR	106.67	84.97	95.82	8	24.0
37	NRRMKWKK	17.42	11.25	14.33	5	10.5
38	RRRRRRRW	83.21	64.01	73.61	7	17.6
39	EEEAAKKK	19.06	4.20	11.63	0	17.0
40	GRKRKKRT	12.77	9.56	11.17	6	17.6
41	RNRSRHRR	8.22	4.91	6.57	6	15.0
42	MIIYRDLI	30.46	24.76	27.61	0	-4.8
43	IYRDLISH	7.75	15.37	11.56	1	-1.9
44	WRFKWRFK	91.52	88.32	89.92	4	0.2
45	KERKKRRR	15.92	21.93	18.93	6	24.0
46	RIFIRIGC	29.72	23.09	26.41	2	-2.9
47	RKKRRQRRR	103.80	41.13	72.47	8	24.2
48	RRRQRRKKR	51.88	30.95	41.42	8	24.2
49	RKKRRARRR	51.62	38.01	44.81	8	23.5
50	RRRRRRRRR	57.96	35.39	46.67	9	27.0
51	QNRRMKWKK	16.67	16.91	16.79	5	10.7
52	RRRERRAEK	11.68	8.49	10.09	4	23.5
53	KCPSRRPKR	30.30	35.02	32.66	5	14.3
54	KGKKIFIMK	54.93	57.09	56.01	4	4.6
55	CGNKRTRGC	10.70	13.14	11.92	3	6.8
56	MIIYRDLIS	8.19	6.19	7.19	0	-4.5
57	IIYRDLISH	4.30	12.14	8.22	1	-3.7
58	AIIYRDLIS	3.14	3.41	3.28	0	-3.7
59	MAIYRDLIS	3.91	1.92	2.92	0	-3.2
60	MIAYRDLIS	9.17	6.53	7.85	0	-3.2
61	MIIARDLIS	9.25	5.48	7.36	0	-2.7
62	MIIYADLIS	15.28	18.71	16.99	-1	-8.0
63	MIIYRALIS	50.68	26.71	38.70	1	-8.0
64	MIIYRDAIS	26.24	25.81	26.03	0	-3.2
65	MIIYRDLAS	11.07	6.38	8.73	0	-3.2
66	MIIYRDLIA	16.88	9.37	13.12	0	-5.3
67	GRKKRRQRRR	32.48	26.09	29.29	8	24.2
68	FQNRRMKWKK	25.30	27.83	26.57	5	8.2
69	RQARRNRRRC	68.84	68.37	68.60	6	16.9
70	MLLLTRRRST	32.67	27.88	30.27	3	1.8
71	PRPLPFPRPG	3.08	2.92	3.00	2	1.7
72	KRIPNKKPKK	25.28	35.53	30.40	6	16.4
73	RRIPNRRPRR	18.36	20.73	19.54	6	16.4
74	KKPTIKTTKK	4.26	2.43	3.34	5	12.0
75	KKTTTKPTKK	0.40	4.05	2.23	5	13.4
76	DRDRDRDRDR	0.40	-0.30	0.05	0	30.0
77	RTRRNRRRVR	23.25	22.19	22.72	7	19.3
78	WRFKKSKRKV	50.28	32.83	41.56	6	10.9
79	GKKKKKKKKK	10.69	7.86	9.28	9	27.0
80	GRRRRRERNK	10.91	4.51	7.71	6	24.2
81	ARAAARQARA	1.70	1.48	1.59	3	6.2
82	MIIYRDLISH	5.54	9.19	7.36	1	-5.0
83	MIIYRDKKSH	6.25	5.53	5.89	3	4.6

No.	Sequence	RFU 1	RFU 2	Mean RFU	Net charge pH 7.4	Hydro-pathy score
84	MIIFRDLISH	13.43	8.18	10.81	1	-5.2
85	MIISRDLISH	2.72	2.04	2.38	1	-2.4
86	QIISRDLISH	3.03	-0.49	1.27	1	-0.9
87	CIISRDLISH	13.60	12.30	12.95	1	-2.1
88	MIIRRDLISE	5.03	1.23	3.13	0	3.8
89	MIIYRAEISH	14.89	17.40	16.15	1	-3.7
90	MIIYARRAEE	15.23	11.18	13.21	0	3.8
91	LIIFRILISH	50.76	47.53	49.15	2	-10.5
92	RIFIHFRIGC	46.60	51.34	48.97	3	-5.9
93	CWKKKKKKKK	98.95	86.21	92.58	8	19.6
94	GRKKRRQRRRC	133.95	86.52	110.23	8	23.2
95	RRRRRRRRRRR	25.87	26.21	26.04	11	33.0
96	WFQNRRMKWKK	52.39	45.57	48.98	5	4.8
97	GKKKKRKREKL	2.95	3.21	3.08	7	25.2
98	KRIPNKKPGKK	0.74	0.03	0.39	6	16.4
99	KTIPSNKPKKK	1.59	5.07	3.33	5	13.3
100	NRARRNRRRVR	19.22	11.63	15.43	7	19.4
101	YGRKKRRQRRR	58.96	42.49	50.73	8	21.9
102	PNTRVRPDVSF	2.10	3.93	3.01	1	3.6
103	PARAARRAARR	9.02	3.63	6.33	5	12.5
104	YPRAARRAARR	12.71	28.10	20.40	5	10.7
105	YRRAARRAARA	24.78	35.92	30.35	5	10.2
106	YGRAARRAARR	12.04	17.87	14.96	5	10.7
107	YAREARRAARR	7.64	15.56	11.60	4	13.7
108	YEREARRAARR	18.66	3.96	11.31	3	17.2
109	YKRAARRAARR	19.82	32.35	26.09	6	13.7
110	YARKARRAARR	36.39	30.55	33.47	6	13.7
111	YKRKARRAARR	26.17	40.64	33.40	7	17.2
112	YGRRARRAARR	33.71	34.46	34.09	6	14.2
113	YGRRARRRARR	53.59	42.54	48.06	7	17.7
114	YGRRARRRRRR	58.08	73.04	65.56	8	21.2
115	YGRRRRRRRRR	51.95	63.81	57.88	9	24.7
116	YRRRRRRRRRR	31.85	60.05	45.95	10	27.7
117	YARAAARQARA	5.25	7.27	6.26	3	3.9
118	RRRQRRKKRGY	64.06	76.75	70.41	8	21.9
119	YARAARRAARR	36.30	40.72	38.51	5	10.2
120	MIIYRDLISKK	21.52	17.40	19.46	2	1.5
121	LIIFRILISHK	137.84	137.03	137.43	3	-7.5
122	LIIFRILISHR	56.41	46.19	51.30	3	-7.5
123	RILQQLLFIHF	37.23	32.46	34.84	2	-11.1
124	IWFQNRRMKWKK	76.44	64.47	70.46	5	3.0
125	YGRKKKRRQRRR	66.52	92.00	79.26	9	24.9
126	PLSSIFSRIGDP	6.76	5.32	6.04	0	-1.0
127	PSSSSSSRIGDP	18.46	13.53	15.99	0	6.0
128	TSPLNIHNGQKL	3.11	9.11	6.11	2	-2.4
129	GRKKRRQRRRPP	33.22	23.16	28.19	8	24.2
130	KRIPNKKPGKKT	5.50	7.85	6.67	6	16.0
131	KPRSKNPPKKPK	29.44	3.67	16.55	6	18.5
132	TRRSKRRSHRKF	24.37	34.90	29.64	8	18.2
133	TFPQTAIGVGAP	2.99	9.92	6.46	0	-7.4

No.	Sequence	RFU 1	RFU 2	Mean RFU	Net charge pH 7.4	Hydro-pathy score
134	KRIIQRILSRNS	15.41	15.41	15.41	4	5.8
135	KRIHPRLTRSIR	8.18	8.97	8.58	6	9.0
136	PPRLRKRRQLNM	9.19	15.86	12.53	5	10.5
137	PIRRRKKLRRLK	31.20	38.26	34.73	8	18.6
138	RRQRRTSKLMKR	10.05	16.44	13.25	7	18.0
139	MHKRPTTPSRKM	6.36	6.21	6.28	5	8.4
140	RQRSRRRPLNIR	9.23	14.15	11.69	6	15.1
141	RIRMIQNLIKKT	19.79	24.55	22.17	4	3.5
142	SRRKRQRSNMRI	10.00	9.14	9.57	6	15.9
143	QRIRKSKISRTL	11.23	12.81	12.02	5	10.0
144	PSKRLLHNNLRR	12.29	4.26	8.28	5	6.8
145	HRHIRRQSLIML	22.17	27.48	24.83	5	0.0
146	PQNRLQIRRHSK	19.27	14.71	16.99	5	8.8
147	PPHNRIQRRLNM	3.59	2.12	2.85	4	4.2
148	SMLKRNHSTSNR	13.89	4.16	9.02	4	6.3
149	GSRHPSLIIPRQ	7.41	5.02	6.21	3	0.9
150	SPMQKTMNLPPM	18.22	13.69	15.95	1	-2.4
151	NKRILIRIMTRP	77.94	87.01	82.48	4	3.3
152	LTRNYEAWVPTP	5.57	10.39	7.98	0	-4.1
153	YSHIATLPFTPT	6.62	3.56	5.09	1	-10.3
154	SYIQRTPSTTLP	45.16	20.00	32.58	1	-3.3
155	AVPAENALNNPF	26.30	5.20	15.75	-1	-3.7
156	SFHQFARATLAS	31.01	2.94	16.98	2	-5.4
157	QSPTDFTFPNPL	13.35	4.56	8.96	-1	-3.9
158	HFAAWGGWSLVH	18.56	6.63	12.59	2	-14.3
159	HIQLSPFSQSWR	28.53	11.26	19.90	2	-5.7
160	LTMPSDLQPVLW	11.88	8.61	10.24	-1	-8.5
161	FQPYDHPAEVSY	3.34	16.86	10.10	-1	-3.1
162	FDPFFWKYSPRD	61.31	60.58	60.94	0	-0.9
163	FAPWDTASFMLG	3.45	3.29	3.37	-1	-9.6
164	FTYKNFFWLPEL	53.26	47.29	50.28	0	-11.0
165	SATGAPWKMWVR	13.23	10.05	11.64	2	-4.7
166	SLGWMLPFSPPF	11.69	6.52	9.11	0	-12.7
167	SHAFTWPTYLQL	14.60	6.36	10.48	1	-13.1
168	SHNWLPLWPLRP	88.18	23.78	55.98	2	-9.2
169	SWLPYPWHVPSS	4.82	3.94	4.38	1	-12.0
170	SWWTPWHVHSES	7.06	3.01	5.04	1	-9.2
171	SWAQHLSLPPVL	18.25	7.05	12.65	1	-10.5
172	SSSIFPPWLSFF	48.35	23.97	36.16	0	-13.3
173	LNVPPSWFLSQR	22.23	8.03	15.13	1	-7.0
174	LDITPFLSLTLP	58.45	31.89	45.17	-1	-9.0
175	LPHPVLHMGPLR	26.53	6.66	16.59	3	-6.2
176	VSKQPYYMWNGN	8.31	5.59	6.95	1	-6.9
177	NYTTYKSHFQDR	2.09	12.39	7.24	2	1.3
178	AIPNNQLGFPFK	6.54	3.81	5.18	1	-5.5
179	NIENSTLATPLS	2.27	0.87	1.57	-1	-2.7
180	YPYDANHTRSPT	10.05	5.21	7.63	1	0.1
181	DPATNPGPHFPR	8.88	-0.68	4.10	1	2.3
182	TLPSPLALLTVH	24.95	12.79	18.87	1	-10.2
183	HPGSPFPPEHRP	6.55	0.51	3.53	2	2.8

No.	Sequence	RFU 1	RFU 2	Mean RFU	Net charge pH 7.4	Hydro-pathy score
184	TSHTDAPPARSP	0.97	2.06	1.51	1	4.3
185	MTPSSLSTLPWP	6.75	3.89	5.32	0	-8.2
186	VLGQSGYLMPMR	2.24	20.64	11.44	1	-6.5
187	QPIIITSPYLPS	3.58	3.22	3.40	0	-9.1
188	TPKTMTQTYDFS	1.73	0.34	1.04	0	-1.2
189	NSGTMQSASRAT	5.74	1.64	3.69	1	1.2
190	QAASRVENYMHR	1.60	0.52	1.06	2	3.1
191	HQHKPPPLTNNW	6.56	6.79	6.68	3	-3.0
192	SNPWDSLLSVST	1.92	2.77	2.35	-1	-4.5
193	KTIEAHPPYYAS	8.11	4.36	6.23	1	-2.0
194	EPDNWSLDFPRR	2.96	3.00	2.98	-1	7.8
195	WRFKWRFKWRFK	127.09	181.82	154.46	6	0.3
196	APWHLSSQYSRT	4.34	5.13	4.73	2	-4.8
197	GRKGKHKRKKLP	13.68	13.97	13.82	8	18.7
198	GRRRRKRLSHRT	17.59	23.94	20.76	8	18.6
199	SQMTRQARRLYV	6.98	12.55	9.76	3	1.9
200	MIIYRALISHKK	83.94	80.04	81.99	4	-2.5
201	MIIYRIAASHKK	42.66	54.46	48.56	4	-1.2
202	MIIFRIAASHKK	111.63	129.08	120.36	4	-1.4
203	MIIFRALISHKK	154.43	237.03	195.73	4	-2.7
204	MIIFRAAASHKK	75.10	58.76	66.93	4	-0.1
205	FIIFRIAASHKK	248.98	294.27	271.62	4	-2.6
206	LIIFRIAASHKK	168.75	243.30	206.03	4	-1.9
207	WIIFRIAASHKK	171.44	184.39	177.92	4	-3.5
208	WIIFRAAASHKK	92.58	90.13	91.36	4	-2.2
209	WIIFRALISHKK	194.27	176.52	185.39	4	-4.8
210	MIIFRIAAYHKK	168.20	182.24	175.22	4	-4.0
211	WIIFRIAAYHKK	191.01	186.52	188.76	4	-6.1
212	MIIFRIAATHKK	225.35	172.63	198.99	4	-2.1
213	WIIFRIAATHKK	289.67	256.44	273.05	4	-4.2
214	MIIFKIAASHKK	91.97	95.79	93.88	4	-1.4
215	WIIFKIAASHKK	127.04	147.00	137.02	4	-3.5
216	MIIFAIAASHKK	89.85	98.48	94.17	3	-4.9
217	LIIFRILISHKK	153.55	150.97	152.26	4	-4.5
218	MIIFRILISHKK	131.43	153.29	142.36	4	-4.0
219	LIIFRILISHRR	165.30	75.39	120.35	4	-4.5
220	LIIFRILISHHH	96.79	100.62	98.71	4	-11.5
221	LIIFAIAASHKK	127.14	150.12	138.63	3	-5.4
222	LIIFAILISHKK	31.45	34.39	32.92	3	-8.0
223	GRKKRRQRRRPPQ	19.55	39.24	29.40	8	24.4
224	GRKKRRQRRPPQC	81.88	122.62	102.25	7	20.4
225	RQIKIWFQNRRMK	53.44	78.98	66.21	5	4.8
226	KIWFQNRRMKWKK	46.19	56.97	51.58	6	6.0
227	GRKKRRQRRRPWQ	50.70	120.37	85.54	8	21.0
228	ALWKTLLKKVLKA	61.21	87.24	74.23	4	-1.5
229	GRRRRRRRRRPPQ	59.77	101.83	80.80	9	27.2
230	KRVSRNKSEKKRR	13.70	23.48	18.59	7	26.3
231	RRHHCRSKAKRSR	67.58	128.25	97.91	9	19.1
232	GPFHFYQFLFPPV	38.20	39.46	38.83	1	-15.9
233	GSPWGLQHHPPRT	1.53	2.01	1.77	3	-3.1

No.	Sequence	RFU 1	RFU 2	Mean RFU	Net charge pH 7.4	Hydro-pathy score
234	CVQWSLLRGYQPC	49.80	55.71	52.75	1	-9.1
235	KLIKGRTPIKFGK	5.89	6.87	6.38	5	6.7
236	VRRFLVTLRIRRA	178.62	171.40	175.01	5	3.2
237	RVRVFVVHIPRLT	261.48	288.41	274.94	4	-4.0
238	GYGRKKRRQRRRG	66.84	89.27	78.06	8	21.9
239	SAETVESCLAKSH	38.07	50.56	44.32	0	3.7
240	YRFKYRFKYRLFK	126.26	206.96	166.61	6	1.8
241	RQIKIWFQNRRMKW	89.70	156.39	123.04	5	1.4
242	IKIWFQNRRMKWKK	177.08	198.09	187.58	6	4.2
243	GRKKRRQRRRPPQC	155.42	186.67	171.04	8	23.4
244	SRRARRSPRHLGSG	4.67	7.46	6.07	6	13.1
245	KKPGKKTTTKPTKK	12.05	20.65	16.35	7	19.4
246	ERERERERERERER	2.79	1.37	2.08	0	42.0
247	WRWRWRWRWRWRWR	53.30	60.30	56.80	7	-2.8
248	GRRRRATAKYRTAH	34.08	36.15	35.11	7	12.9
249	RLSGMNEVLSFRWL	40.88	19.05	29.97	1	-4.3
250	RQIKIWFQNRRMKWK	258.78	230.35	244.57	6	4.4
251	QIKIWFQNRRMKWKK	194.51	179.41	186.96	6	4.4
252	TRRQRTRRARRNRGC	129.11	101.52	115.32	8	22.1
253	KWFETWFTEWPKKRK	65.68	68.73	67.21	3	5.0
254	PPKKSAQCLRYKKPE	16.34	15.13	15.73	4	12.9
255	DPVDTPNPTRRKPGK	5.34	12.67	9.01	2	15.9
256	GKRKKKGKLGKKRDP	6.85	10.49	8.67	8	28.2
257	KLWMRWYSPTTRRYG	98.47	106.49	102.48	4	-3.0
258	DSLKSYWYLQKFSWR	61.66	73.33	67.50	2	-4.4
259	RTLVNEYKNTLKFSK	8.99	10.74	9.86	3	5.0
260	IPSRWKDQFWKRWHY	106.20	125.84	116.02	4	-1.8
261	GYGNCRHFKQKPRRD	70.25	67.03	68.64	5	12.1
262	KNAWKHSSCHHRHQI	25.42	25.86	25.64	7	1.3
263	RVREWWYTITLKQES	45.36	48.53	46.94	1	0.5
264	QQHLLIAINGYPRYN	24.40	25.13	24.77	2	-9.0
265	WKCRRQCFRVLHHWN	90.28	100.43	95.36	6	-3.2
266	RLWMRWYSPTTRRYG	91.42	117.40	104.41	4	-3.0
267	KLWMRWYSATTRRYG	122.95	129.11	126.03	4	-3.5
268	KLWMRWYSPWTRRYG	132.74	106.38	119.56	4	-6.0
269	RLWMRWYSPWTRRYG	36.06	32.07	34.06	4	-6.0
270	RAWMRWYSPTTRRYG	29.62	28.20	28.91	4	-1.7
271	RLAMRWYSPTTRRYG	65.58	68.17	66.87	4	-0.1
272	RLWARWYSPTTRRYG	57.53	72.85	65.19	4	-2.2
273	RLWMRWASPTTRRYG	84.25	82.65	83.45	4	-1.2
274	RLWMRWYAPTTRRYG	75.85	121.00	98.42	4	-3.8
275	RLWMRWYSPATRRYG	88.23	126.61	107.42	4	-3.1
276	RLWMRWYSPTARRYG	76.93	101.02	88.98	4	-3.1
277	RLWMRWYSPTTRRAG	162.13	177.12	169.63	4	-1.2
278	RLWMRWYSPTTRRYA	134.28	162.69	148.49	4	-3.5
279	RLFMRFYSPTTRRYG	90.44	95.60	93.02	4	-1.2
280	RLIMRIYSPTTRRYG	93.28	95.18	94.23	4	0.2
281	RLVMRVYSPTTRRYG	145.19	137.49	141.34	4	0.8
282	RLYMRYYSPTTRRYG	70.93	99.63	85.28	4	-0.8
283	RRIRPRPPRLPRPRP	29.31	30.64	29.97	7	17.4

No.	Sequence	RFU 1	RFU 2	Mean RFU	Net charge pH 7.4	Hydro-pathy score
284	RQLRIAGRRLRGRSR	18.40	32.01	25.20	7	15.6
285	KSICKTIPSNKPKKK	17.81	23.37	20.59	6	13.8
286	QTRRRERRAEKQAQW	12.87	13.15	13.01	4	19.8
287	KFHTFPQTAIGVGAP	5.12	2.69	3.91	2	-7.4
288	FLGKKFKKYFLQLLK	298.36	283.51	290.93	5	-1.8
289	RVIRWFQNKRCKDKK	129.42	140.33	134.88	6	14.2
290	RVIRWFQNKRSKDKK	76.95	64.97	70.96	6	15.5
291	CWKKKKKKKKKKKKK	175.27	145.30	160.28	13	34.6
292	GKRRRRATAKYRSAH	20.15	27.54	23.85	8	16.6
293	TRNKRNRIQEQLNRK	5.69	1.60	3.65	5	18.0
294	RILQQLLFIHFRIGC	80.06	72.19	76.13	3	-10.9
295	FITKALGISYGRKKRR	49.25	71.45	60.35	6	7.2
296	KLALKLALKALKAALK	545.23	411.56	478.40	5	1.7
297	AQIKIWFQNRRMKWKK	142.95	137.50	140.22	6	3.9
298	RAIKIWFQNRRMKWKK	75.31	95.10	85.21	7	6.7
299	RQAKIWFQNRRMKWKK	61.10	98.25	79.67	7	8.7
300	RQIAIWFQNRRMKWKK	164.31	141.04	152.67	6	3.9
301	RQIKAWFQNRRMKWKK	53.46	69.23	61.35	7	8.7
302	RQIKIAFQNRRMKWKK	121.17	124.24	122.70	7	10.3
303	RQIKIWAQNRRMKWKK	77.02	75.93	76.48	7	9.4
304	RQIKIWFANRRMKWKK	176.15	156.11	166.13	7	6.7
305	RQIKIWFQARRMKWKK	188.39	174.83	181.61	7	6.7
306	RQIKIWFQNARMKWKK	75.87	130.88	103.37	6	3.9
307	RQIKIWFQNRAMKWKK	108.70	125.54	117.12	6	3.9
308	RQIKIWFQNRRAKWKK	157.08	158.96	158.02	7	8.2
309	RQIKIWFQNRRMKAKK	120.43	125.54	122.98	7	10.3
310	RQIKIWFPNRRMKWKK	137.08	141.77	139.42	7	7.2
311	RVIRVWFQNKRCKDKK	116.51	121.08	118.79	6	12.7
312	GRRHHCRSKAKRSRHH	74.92	78.91	76.92	11	18.1
313	SARHHCRSKAKRSRHH	44.46	47.69	46.07	10	14.9
314	SRAHHCRSKAKRSRHH	44.97	47.65	46.31	10	14.9
315	SRRAHCRSKAKRSRHH	55.36	60.31	57.83	10	18.4
316	SRRHACRSKAKRSRHH	60.63	79.99	70.31	10	18.4
317	SRRHHARSKAKRSRHH	10.17	12.49	11.33	11	18.9
318	SRRHHCRAKAKRSRHH	69.53	69.01	69.27	11	17.6
319	SRRHHCRSAAKRSRHH	58.01	43.24	50.63	10	14.9
320	SRRHHCRSKAARSRHH	50.32	59.45	54.88	10	14.9
321	SRRHHCRSKAKASRHH	49.04	46.18	47.61	10	14.9
322	SRRHHCRSKAKRARHH	59.43	63.51	61.47	11	17.6
323	SRRHHCRSKAKRSAHH	47.11	51.72	49.41	10	14.9
324	RQIKIFFQNRRMKFKK	208.83	212.46	210.65	7	9.2
325	RQIRIWFQNRRMRWRR	106.92	101.29	104.11	7	7.4
326	RKKRRRESRKKRRRES	65.81	67.40	66.60	10	42.6
327	LRRERQSRLRRERQSR	25.48	95.20	60.34	6	27.4
328	KKWKMRRNQFWIKIQR	81.81	82.47	82.14	7	7.4
329	RQPKIWFPNRRKPWKK	58.45	65.64	62.04	7	10.3
330	CSIPPEVKFNPFVYLI	102.64	66.08	84.36	0	-10.2
331	GLRKRLRKFRNKIKEK	35.52	52.25	43.88	8	22.3
332	RRWRRWWRRWWRRWRR	75.17	33.28	54.22	10	9.6
333	RQIKIWFQNMRRKWKK	187.38	203.15	195.27	7	7.4

No.	Sequence	RFU 1	RFU 2	Mean RFU	Net charge pH 7.4	Hydro-pathy score
334	GRQLRIAGKRLEGRSK	6.04	3.15	4.60	5	15.6
335	GRQLRIAGKRLRGRSK	28.39	23.67	26.03	7	15.6
336	GRQLRIAGRRLRGRSR	15.47	16.04	15.76	7	15.6
337	GRQLRRAGRRLRGRSR	18.24	26.40	22.32	8	20.4
338	GRQLRIAGRRLRRRSR	20.34	34.59	27.46	8	18.6
339	GRQLRRAGRRLRRRSR	28.12	41.03	34.57	9	23.4
340	KRPAAIKKAGQAKKKK	9.43	19.29	14.36	8	20.4
341	YIVLRRRRKRVNTKRS	160.77	228.77	194.77	8	15.2
342	KTVLLRKLLKLLVRKI	899.75	811.38	855.56	6	2.0
343	LLKKRKVVRLIKFLLK	449.07	421.53	435.30	7	4.7
344	KKICTRKPRFMSAWAQ	93.11	86.68	89.89	5	4.1
345	RHIKIWFQNRRMKWKK	124.87	161.78	143.32	8	6.7
346	RLWRALPRVLRRLLRP	175.20	204.40	189.80	6	3.6
347	KLWSAWPSLWSSLWKP	110.15	97.57	103.86	2	-12.3
348	AAVALLPAVLLALLAK	160.27	162.37	161.32	1	-13.8
349	GKHRHERGHHRDRRER	13.62	7.55	10.58	8	28.0
350	RQIKIFFQNRRMKWKK	253.88	224.34	239.11	7	8.3
351	YTAIAWVKAFIRKLRK	414.26	388.37	401.31	5	-2.0
352	AAVALLPAVLLALLAP	119.74	144.08	131.91	0	-16.8
353	RQIKIWFQNRRMKWKK	205.31	154.16	179.74	7	7.4
354	GKINLKALAALAKKIL	127.94	109.09	118.51	4	-0.6
355	TRQARRNRRRRWRERQR	118.36	129.32	123.84	9	29.3
356	RRRRNRTRRNRRRVRGC	88.28	58.25	73.27	11	30.5
357	GRPRESGKKRKRKRLKP	27.63	33.18	30.40	9	31.5
358	GKRKKKGKLGKKRPRSR	57.48	49.62	53.55	11	31.5
359	RKKRRRESRKKRRRESC	111.40	104.67	108.04	10	41.6
360	DRDDRDDRDDRDDRDDR	1.99	2.12	2.05	-5	51.0
361	DRRRRGSRPSGAERRRR	12.99	14.01	13.50	7	33.1
362	RAGLQFPVGRVHRLLRK	15.97	12.96	14.46	6	3.3
363	GKKTNLFSALIKKKKTA	11.31	22.57	16.94	6	8.8
364	GKKKRKLSNRESAKRSR	5.81	9.52	7.67	8	28.8
365	IAWVKAFIRKLRKGPLG	175.17	142.95	159.06	5	-0.6
366	CRQIKIWFQNRRMKWKK	111.51	101.59	106.55	7	6.4
367	KGEGAAVLLPVLLAAPG	3.04	8.58	5.81	0	-6.2
368	MGLGLHLLVLAAALQGA	34.76	28.92	31.84	1	-15.9
369	GALFLGWLGAAGSTMGA	7.35	9.68	8.52	0	-14.7
370	RILQQLLFIHFRIGCRH	95.18	80.35	87.76	5	-8.4
371	LGISYGRKKRRQRRRPPQ	70.36	68.94	69.65	8	18.8
372	KLALKLALKAWKAALKLA	331.37	287.31	309.34	5	-2.2
373	KALAKALAKLWKALAKAA	351.80	339.37	345.58	5	0.4
374	KALKKLLAKWLAAAKALL	280.41	297.33	288.87	5	-2.2
375	KLAAALLKKWKKLAAALL	322.69	349.10	335.89	5	-2.2
376	KALAALLKKWAKLLAALK	268.72	271.18	269.95	5	-2.2
377	KALAALLKKLAKLLAALK	204.44	233.77	219.11	5	-0.6
378	KLALKLALKALQAALQLA	294.36	255.99	275.18	3	-6.2
379	KLALQLALQALQAALQLA	85.34	69.54	77.44	1	-11.8
380	QLALQLALQALQAALQLA	122.23	76.78	99.51	0	-14.6
381	LKTLATALTKLAKTLTTL	229.69	208.17	218.93	3	-5.7
382	LLKTTALLKTTALLKTTA	60.19	46.42	53.31	3	-5.7
383	LLKTTELLKTTELLKTTE	16.42	20.95	18.69	0	4.8

No.	Sequence	RFU 1	RFU 2	Mean RFU	Net charge pH 7.4	Hydro-pathy score
384	KALKLKLALALLAKLKLA	327.00	270.27	298.64	5	-1.9
385	CRQIKIWFPNRRMKWKKC	89.08	87.68	88.38	7	5.2
386	RKKRRRESRRARRSPRHL	54.22	83.19	68.70	11	33.8
387	LLIILRRRIRKQAHAHSK	228.82	222.63	225.72	8	5.7
388	LLIILARRIRKQAHAHSK	161.55	164.49	163.02	7	2.2
389	LLIILRARIRKQAHAHSK	220.49	200.51	210.50	7	2.2
390	LLIILRRAIRKQAHAHSK	196.23	222.87	209.55	7	2.2
391	LLIILRRRIRAQAHAHSK	219.10	197.64	208.37	7	2.2
392	LLIILRRRIRKAAHAHSK	246.09	256.69	251.39	8	5.0
393	LLIILRRRIRKQAHAASK	238.90	224.92	231.91	7	5.7
394	LLIILRRRIRKQAHAHAK	238.46	224.29	231.37	8	4.9
395	LLIILRRRIRKQAHAHSA	259.37	231.11	245.24	7	2.2
396	VRLPPPVRLPPPVRLPPP	18.90	14.51	16.70	3	-0.9
397	VKLPPPVKLPPPVKLPPP	31.52	21.65	26.59	3	-0.9
398	DFNKFHTFPQTAIGVGAP	11.64	10.05	10.84	1	-6.7
399	LGLLLRHLRHHSNLLANI	222.32	147.05	184.69	5	-9.7
400	GKRVAKRKLIEQNRERRR	16.79	36.26	26.52	7	27.8
401	GRKLKKKKNEKEDKRPRT	20.66	13.48	17.07	7	37.0
402	GRRERNKMAAAKCRNRRR	65.39	107.82	86.61	8	26.6
403	GKRARNTEAARRSRARKL	31.95	15.06	23.50	7	23.3
404	TAKTRYKARRAELIAERR	13.72	29.91	21.81	5	18.3
405	KLALKLALKALKAALKLA	425.81	455.01	440.41	5	-0.6
406	NAKTRRHERRRKLAIERGC	38.44	55.58	47.01	7	23.7
407	SRRARRSPRESGKKRKRKR	38.35	67.00	52.67	11	39.4
408	LLIILRRRIARKQAHAHSK	218.54	240.27	229.41	8	5.2
409	CVKRGLKLRHVRPRVTRDV	95.78	75.14	85.46	7	12.5
410	KRIPNKKPGKKTTTKPTKK	9.74	11.26	10.50	9	23.8
411	KLPCRSNTFLNIFRRKKPG	149.71	144.72	147.21	6	6.9
412	KKKKKKKKKKKKKKKKKKK	714.73	549.28	632.01	19	57.0
413	KCFQWQRNMRKVRGPPVSC	72.37	67.44	69.90	5	3.7
414	ALWKTLLKKVLKAPKKKRKV	138.84	148.90	143.87	9	12.0
415	PKKKRKVALWKTLLKKVLKA	465.84	315.77	390.81	9	12.0
416	VKRKKKPALWKTLLKKVLKA	847.15	1003.69	925.42	9	12.0
417	AYRIKPTFRRLKWKYKGKFW	185.47	149.26	167.36	8	3.1
418	HARIKPTFRRLKWKYKGKFW	125.63	82.16	103.89	9	4.9
419	HYRIKPTARRLKWKYKGKFW	85.45	74.67	80.06	9	5.1
420	HYRIKPTFRRLAWKYKGKFW	143.87	102.01	122.94	8	-0.4
421	HYRIKPTFRRLKWKYKGKFA	83.07	96.36	89.71	9	6.0
422	GLWRALWRLLRSLWRLLWRA	39.58	17.74	28.66	5	-11.9
423	GLWWRLWWRLRSWFRLWFRA	27.87	18.53	23.20	5	-17.8
424	GLWRALWRLLRSLWRLLWKA	54.34	42.87	48.60	5	-11.9
425	GLWRALWRALRSLWKLKRKV	413.88	355.41	384.64	7	-0.4
426	GLWRALWRGLRSLWKLKRKV	412.88	282.45	347.66	7	0.1
427	GLWRALWRGLRSLWKKKRKV	378.22	372.12	375.17	8	4.9
428	WRFKAAVALLPAVLLALLAP	48.41	54.31	51.36	2	-16.7
429	CWKKKKKKKKKKKKKKKKKK	447.98	387.89	417.94	18	49.6
430	GLFEALLELLESLWELLLEA	110.53	67.50	89.02	-5	-7.8
431	GLFKALLKLLKSLWKLLLKA	361.81	291.52	326.66	5	-7.8
432	GLFRALLRLLRSLWRLLLRA	107.93	67.31	87.62	5	-7.8
433	RILQQLLFIHFRIGCRHSRI	98.43	90.93	94.68	6	-6.9

No.	Sequence	RFU 1	RFU 2	Mean RFU	Net charge pH 7.4	Hydro-pathy score
434	KLALKLALKALKAALKLAGC	217.31	203.08	210.19	5	-1.6
435	KMTRAQRRAAARRNRWTARGC	98.17	100.68	99.43	8	15.4
436	KLTRAQRRAAARKNKRNTRGC	107.93	107.44	107.68	9	22.0
437	KETWWETWWTEWSQPKKKRKV	94.75	96.37	95.56	3	7.8
438	KETWFETWFTEWSQPKKKRKV	47.66	56.84	52.25	3	9.6
439	AGYLLGKINLKALAALAKKIL	266.77	252.89	259.83	4	-7.0
440	YTQDFNKFHTFPQTAIGVGAP	8.03	12.55	10.29	1	-9.2
441	GLWRALWRALWRSLWKLKRKV	291.93	252.26	272.09	7	-3.8
442	GLWRALWRALWRSLWKLKWKV	205.86	205.36	205.61	6	-10.2
443	GLWRALWRALWRSLWKSKRKV	288.44	250.82	269.63	7	-1.7
444	GLWRALWRALWRSLWKKKRKV	324.82	261.27	293.05	8	1.0
445	ASMWERVKSIIKSSLAAASNI	24.03	30.32	27.17	2	-1.7
446	RQARRNRRRALWKTLLKKVLKA	492.51	409.43	450.97	10	16.4
447	GAYDLRRRERQSRLRRRERQSR	31.54	30.07	30.80	7	33.6
448	TKRRITPKDVIDVRSVTTEINT	4.49	2.31	3.40	2	12.6
449	TKRRITPKKVIDVRSVTTEINT	12.47	9.40	10.94	4	12.6
450	TKRRITPKDVIDVRSVTTKINT	7.81	4.07	5.94	4	12.6
451	MVRRFLVTLRIRRACGPPRVRV	49.62	38.02	43.82	7	3.9
452	FVTRGCPRRLVARLIRVMVPRR	185.18	129.04	157.11	7	3.9
453	MVTVLFRRLRIRRACGPPRVRV	89.51	65.95	77.73	7	3.9
454	RVTSWLGRQLRIAGKRLEGRSK	92.28	63.50	77.89	6	11.8
455	RVRSWLGRQLRIAGKRLEGRSK	71.96	71.83	71.89	7	15.2
456	KKPGKKTTTKPTKKPTIKTTKK	12.48	11.52	12.00	10	25.4
457	DRRRRGSRPSGAERRRRRAAAA	12.40	15.02	13.71	8	34.1
458	KCFQWQRNMRKVRGPPVSCIKR	104.13	79.26	91.69	7	7.9
459	AAVALLPAVLLALLAPRRRRRR	115.31	100.52	107.91	6	1.2
460	GLGSLLKKAGKKLKQPKSKRKV	41.79	39.15	40.47	9	18.6
461	KCFMWQEMLNKAGVPKLRCARK	82.55	81.78	82.16	5	4.8
462	DTWAGVEAIIRILQQLLFIHFR	86.68	65.88	76.28	1	-12.0
463	GEQIAQLIAGYIDIILKKKKSK	231.55	159.53	195.54	3	5.8
464	FITKALGISYGRKKRRQRRRPPQ	117.46	123.66	120.56	9	16.6
465	MGLGLHLLVLAAALQGAKKKRKV	317.74	277.58	297.66	6	-2.4
466	LIRLWSHLIHIWFQNRRLKWKKK	521.62	329.58	425.60	9	-4.6
467	CGAYDLRRRERQSRLRRRERQSR	69.79	29.32	49.56	7	32.6
468	RRVTSWLGRQLRIAGKRLEGRSK	179.47	112.54	146.00	7	14.8
469	KRIPNKKPGKKTTTKPTKKPTIK	18.91	20.46	19.69	10	24.6
470	FLIFIRVICIVIAKLKANLMCKT	31.61	31.09	31.35	4	-14.9
471	WLRRIKAWLRRIKALNRQLGVAA	1097.27	925.57	1011.42	7	0.3
472	MDAQTRRRERRAEKQAQQWKAAN	16.48	12.16	14.32	4	23.4
473	LNSAGYLLGKINLKALAALAKKIL	467.70	376.56	422.13	4	-8.3
474	LGTYTQDFNKFHTFPQTAIGVGAP	15.89	14.55	15.22	1	-11.4

RFU = relative fluorescence units, RFU1, RFU2 = relative fluorescence units from two independent uptake experiments

Supplementary Table TS2

Mass spectrometry analyzed CPPs equipped with carboxyfluorescein (FAM) at the N-terminus

Sequence length	Peptide sequence	Theoretical mass (Da)	Detected mass (Da)
5	RRRRR	1155.5793	1155.5778
5	VPTLQ	913.3852	913.3853
7	RRRRRRR	1467.7815	1467.7806
8	RNRSRHRR	1493.7132	1493.7124
9	RRRQRRKKR	1695.9289	1695.9270
10	GRKKRRQRRR	1752.9504	1752.9492
11	GRKKRRQRRRC	1855.9595	1855.9563
11	YGRKKRRQRRR	1916.0137	1916.0245
11	YGRRARRAARR	1744.8765	1744.8745
11	RRRQRRKKRGY	1916.0137	1916.0250
12	KRIPNKKPGKKT	1750.9513	1750.9485
12	TRRSKRRSHRKF	1971.0083	1971.0058
12	LIIFRIAASHKK	1752.9346	1752.9327
12	MIIFRILISHKK	1854.9849	1854.9824
13	ALWKTLLKKVLKA	1868.0595	1868.0586
15	QIKIWFQNRRMKWKK	2446.2515	2446.2480
15	DSLKSYWYLQKFSWR	2363.0682	2363.0686
15	KLWMRWYSATTRRYG	2331.0678	2331.0659
15	CWKKKKKKKKKKKKK	2329.3967	2329.3947
16	KLALKLALKALKAALK	2049.2385	2049.2371
16	RQIKIWFQNRRMKWKK	2602.3527	2602.3521
16	RAIKIWFQNRRMKWKK	2545.3312	2545.3286
16	RQIKAWFQNRRMKWKK	2560.3057	2560.3035
16	RQIKIWFANRRMKWKK	2545.3312	2545.3286
16	RQIKIWFQARRMKWKK	2559.3468	2559.3440
16	RQIKIWFQNRRAKWKK	2542.3493	2542.3468
16	RQIKIFFQNRRMKFKK	2524.3309	2524.3276
16	KKWKMRRNQFWIKIQR	2602.3527	2602.3504
16	RQIKIWFQNMRRKWKK	2602.3527	2602.3477
16	YIVLRRRRKRVNTKRS	2457.3612	2457.3579
16	KTVLLRKLLKLLVRKI	2290.4288	2290.4287
16	LLKKRKVVRLIKFLLK	2351.4604	2351.4594
16	RLWRALPRVLRRLLRP	2427.3911	2428.2939
16	RQIKIWFQNRRMKWKK	2602.3526	2603.0069
17	GKKTNLFSALIKKKKTA	2232.2301	2235.4192
17	IAWVKAFIRKLRKGPLG	2309.2831	2310.3536
18	KLALKLALKALKAALKLA	2233.3597	2233.3564
18	KALAKALAKLWKALAKAA	2222.2610	2222.2593
18	KLAAALLKKWKKLAAALL	2306.3549	2307.4468
18	KLALKLALKALQAALQLA	2233.2869	2233.3118
18	LGLLLRHLRHHSNLLANI	2446.3017	2446.3017
20	ALWKTLLKKVLKAPKKKRKV	2732.6616	2733.1622
20	AYRIKPTFRRLKWKYKGKFW	3028.6011	3027.8221
20	GLWRALWRGLRSLWKKKRKV	2893.6127	2893.6052
20	WRFKAAVALLPAVLLALLAP	2489.3870	2489.3792
20	CWKKKKKKKKKKKKKKKKKK	2969.8716	2969.8678
21	AGYLLGKINLKALAALAKKIL	2538.4608	2538.4523

Sequence length	Peptide sequence	Theoretical mass (Da)	Detected mass (Da)
21	YTQDFNKFHTFPQTAIGVGAP	2695.2014	2695.1903
21	GLWRALWRALWRSLWKKKRKV	3093.7077	3093.7045
22	TKRRITPKKVIDVRSVTTEINT	2911.5550	2911.5457
23	MGLGLHLLVLAAALQGAKKKRKV	2771.5667	2771.5738
23	WLRRIKAWLRRIKALNRQLGVAA	3144.7721	3144.7647
23	MDAQTRRRERRAEKQAQQWKAAN	3184.5116	3184.5024
24	LNSAGYLLGKINLKALAALAKKIL	2852.6199	2852.6196

Supplementary Formula F1. Calculation of net charge (Z):

$$Z = \sum_i Ni \frac{10^{pkai}}{10^{pH} + 10^{pkai}} - \sum_j Nj \frac{10^{pH}}{10^{pH} + 10^{pkaj}}$$

pka_i values: Lysine (K) = 10.5, Arginine (R) = 12.4, Histidine (H) = 6; pka_j values: Glutamic acid (E) = 4.25, Aspartic Acid (D) = 3.86, Cysteine (C) = 8.33, Tyrosine (Y) = 10.

Ni: number of individual amino acids with pka_i values; *Nj*: number of individual amino acids with pka_j values

Supplementary Formula F2. Calculation of hydropathy score (H):

$$H = \sum_i Ni\, Hi$$

Ni: number of individual amino acids with respective hydrophilicity value *Hi*

Supplementary Table TS3

Amino acid residues with their respective hydrophilicity values for calculating peptide hydropathy scores.

**Amino acid**	**Hydrophilicity value *Hi***
Arginine (R)	3.0
Aspartic acid (D)	3.0
Glutamic acid (E)	3.0
Lysine (K)	3.0
Serine (S)	0.3
Asparagine (R)	0.2
Glutamine (Q)	0.2
Glycine (G)	0.0
Proline (P)	0.0
Threonine (T)	-0.4
Alanine (A)	-0.5
Histidine (H)	-0.5
Cysteine (C)	-1.0
Methionine (M)	-1.3
Valine (V)	-1.5
Isoleucine (I)	-1.8
Leucine (L)	-1.8
Tyrosine (T)	-2.3
Phenylalanine (F)	-2.5
Tryptophan (W)	-3.4

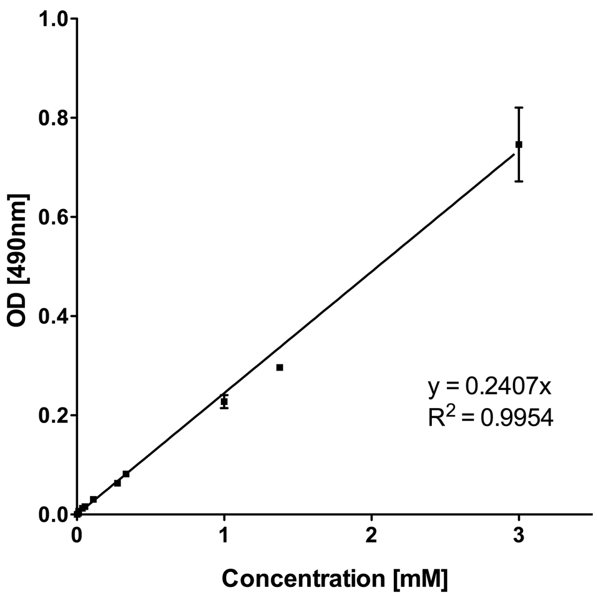

Supplementary Fig. S1. Calibration curve of carboxyfluorescein (FAM) at pH 2.4 for concentration calculation. Measurements were repeated up to three times. (OD: optical density)

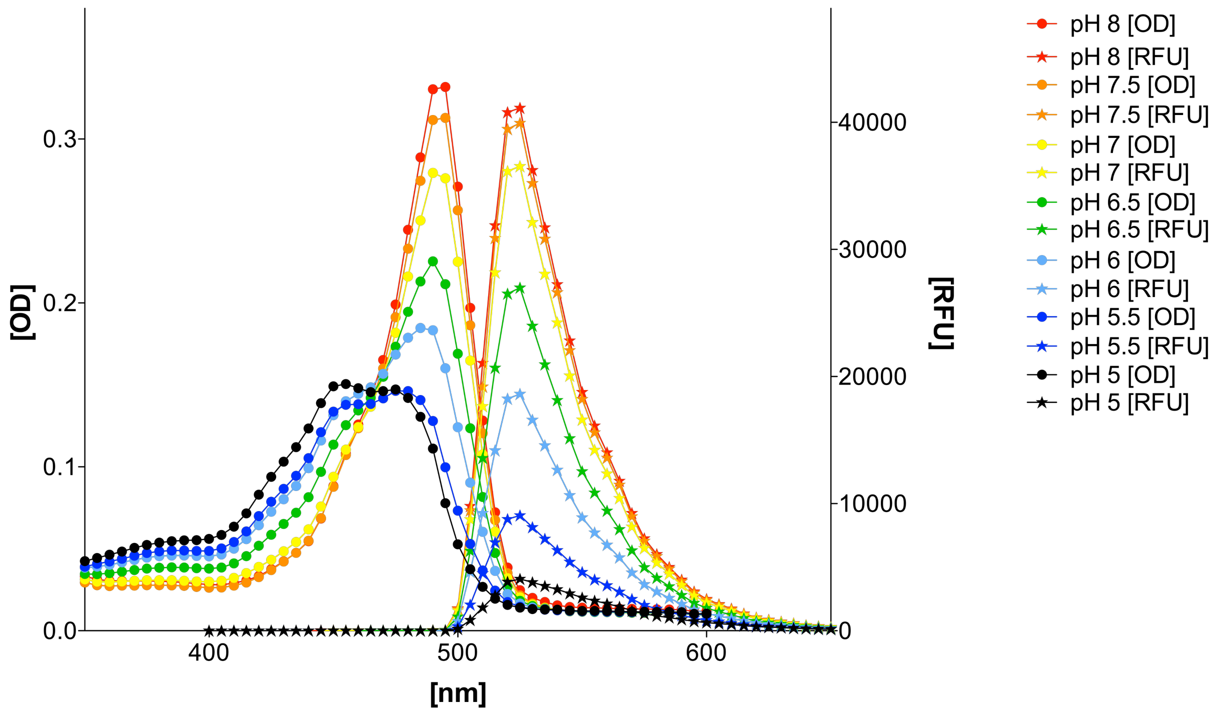

Supplementary Fig. S2. Carboxyfluorescein (FAM) absorbance and fluorescence at different pH values. FAM was dissolved in PBS buffer, pH was adjusted to 8, 7.5, 7, 6.5, 6, 5.5 and 5 respectively. The absorbance spectra ([OD]) of the different solutions were recorded between 350 nm and 600 nm, fluorescence intensities ([RFU], excitation wavelength 495 nm) were measured between 400 nm and 650 nm. The isosbestic point is at 460 nm.
